# Effect of the Addition of Buckwheat Sprouts Modified with the Addition of *Saccharomyces cerevisiae* var. *boulardii* to an Atherogenic Diet on the Metabolism of Sterols, Stanols and Fatty Acids in Rats

**DOI:** 10.3390/molecules27144394

**Published:** 2022-07-08

**Authors:** Marta Molska, Julita Reguła, Anna Grygier, Agata Muzsik-Kazimierska, Magdalena Rudzińska, Anna Gramza-Michałowska

**Affiliations:** 1 Department of Human Nutrition and Dietetics, Faculty of Food Sciences and Nutrition, Poznan University of Life Sciences, 28 Wojska Polskiego Street, 60-637 Poznan, Poland; marta.molska@up.poznan.pl (M.M.); agata.muzsik@up.poznan.pl (A.M.-K.); 2 Departmtent of Food Technology of Plant Origin, Faculty of Food Science and Nutrition, Poznań University of Life Sciences, 28 Wojska Polskiego Street, 60-637 Poznan, Poland; anna.grygier@up.poznan.pl (A.G.); magdalena.rudzinska@up.poznan.pl (M.R.); 3 Department of Gastronomy Sciences and Functional Foods, Faculty of Food Science and Nutrition, Poznań University of Life Sciences, 28 Wojska Polskiego Street, 60-637 Poznan, Poland

**Keywords:** pseudocereal, probiotic yeast, phytocompounds, cholesterol, lipid profile, in vivo, in vitro

## Abstract

The aim of the study was to evaluate the effect of the addition of *Fagopyrum esculentum* Moench buckwheat sprouts modified with the addition of *Saccharomyces cerevisiae* var. *boulardii* to an atherogenic diet on the metabolism of sterols and fatty acids in rats. It was noticed in the study that the group fed with modified sprouts (HFDPRS) had a greater amount of sterols by 75.2%, compared to the group fed on an atherogenic diet (HFD). The content of cholesterol in the liver and feces was lower in the HFDPRS group than the HFD group. In the serum of the HFDPRS group, a more significant amount of the following acids was observed: C18:2 (increase by 13.5%), C20:4 (increase by 15.1%), and C22:6 (increase by 13.1%), compared to the HFDCS group. Regarding the biochemical parameters, it was noted that the group fed the diet with the addition of probiotic-rich sprouts diet had lower non-HDL, LDL-C and CRP ratios compared to the group fed the high-fat diet. The obtained results indicate that adding modified buckwheat sprouts to the diet by adding the probiotic strain of the yeast may have a significant impact on the metabolism of the indicated components in the organism.

## 1. Introduction

Currently, civilization diseases are taking the size of a pandemic. Due to their global spread, they constitute one of the major economic and social health problems. The pathogenesis of most diseases is complex and multifactorial, but a common, important role is played by environmental factors, as well as an inappropriate lifestyle, i.e., an improperly balanced and high-energy diet and limitation of daily physical activity. A diet that consists of highly processed food is low in biologically active ingredients [1]. That is why it is so important to develop novel foods to prevent disease or support therapy and improve the health of patients. Such foods must meet the quality requirements, both chemical and physical, and microbiological requirements, for food products to be safe in its use.

A well-known modifiable risk factor for atherosclerosis is elevated low-density lipoprotein (LDL) cholesterol. It can be lowered by changing the fatty acid composition of the diet. It is also possible to lower LDL cholesterol by consuming products enriched with plant sterols or stanols [2].

Plant sterols (phytosterols) are functional and structural analogs of cholesterol. They are synthesized by plants and are components of plant cell membranes (reducing the fluidity, especially of the surface layer). They are 28 or 29 carbon polycyclic alcohols. Phytosterols have the same polycyclic system as cholesterol, with one hydroxyl group. The difference in construction is in the side chain. Phytosterols are richer with an ethyl or methyl group. They may additionally contain one or two double bonds in this chain. They are divided into the following three main groups: (1) sterols (∆5-sterols, containing a double bond between C5 and C6; (2) ∆7-sterols with a double bond between C7 and C8, less common in nature); (3) stanols (no double bond in the molecule). In their natural state, these compounds exist in free form. They may also appear as sterol or stanol esters of fatty acids, hydroxycinnamic acid, glucose and glycolipids [3,4].

Buckwheat is a pseudocereal that belongs to the *Polygonaceae* family and includes 15 species growing in the temperate climate of Europe and Asia [5]. The most popular varieties are common buckwheat (*Fagopyrum esculentum* Moench) and Tartary buckwheat (*Fagopyrum tataricum* (L.) Gaertn.). Since antiquity, common buckwheat has been cultivated for food and as a medicinal and honey plant [6]. Buckwheat has a healing effect in chronic diseases, such as cancer, diabetes and neurodegenerative diseases, mainly due to bioactive compounds and a well-balanced amino acid pattern [7]. The presence of health-promoting substances in buckwheat makes it more and more often used in various industries, including food, pharmaceutical and cosmetics [8].

The germination process leads to significant changes in the biochemical composition of whole grains. The nitrogen-containing fractions shift towards oligopeptides and free amino acids, and the amino acid composition also changes. In turn, the triacylglycerols begin to hydrolyze and the ratio of saturated to unsaturated fatty acids increases. Starch reserves are mobilized by the action of α-amylase, which corrodes the granular surface and creates holes. The amount of anti-nutritional compounds, i.e., trypsin inhibitor, phytates, and tannins, is significantly reduced. On the other hand, bioactive compounds, such as phenols, phytosterols, folates and GABA, are increased, as a result of which almost all the nutrients in germinating grains are fully available, while various antioxidant compounds are found in higher concentrations. This lays the groundwork for defining sprouts as “functional food” [9].

Consumers are increasingly choosing products that are fresh, highly nutritious, rich in flavor and healthy, including ready-to-eat or ready-to drink foods and beverages. Microbiological trends in food primarily include the use of starter cultures, which can perform many functions (e.g., reduction in toxic compounds, probiotic activity, vitamin production) [10,11]. Probiotics can be used as part of the products that will be their carriers. These are “live strains of carefully selected microorganisms that, when administered in appropriate amounts, provide health benefits to the host”. They can affect both the organoleptic and microbiological quality of food. They are used, for example, in traditional food products, such as fermented milk products. Another possibility of using probiotic microorganisms is to modify the raw material by adding them [12,13].

Functional food “has scientifically proven specific health benefits (health claim) beyond their nutritional properties, but the consumption of its specific composition is not essential to human life. Functional food presentation format is food or derived products such as fortified drinks, juices, milk, yoghurts, margarines, cereals, etc.” [14]. In the study by Świeca et al. 2018, *Lactobacillus plantarum* 299 V legume sprouts were inoculated. This was carried out in order to produce a new functional product. It has been found that legume sprouts are a good source of nutrients [15]. Another study also used sprouts from legumes (lentils and adzuki beans). They were enriched with the addition of *Saccharomyces cerevisiae* var. *boulardii*. The obtained product was characterized by high pro-health and nutritional properties. Taking into account the high content of antioxidant compounds and all aspects related to the documented, beneficial effects of buckwheat components in model and biological systems, a good solution would be to use buckwheat in the production of a new line of food with designed pro-health properties. Buckwheat is gaining more and more interest as a potential functional food. It is noted that buckwheat enriched products, as well as the raw material itself, are associated with health benefits [14,16]. Hence, there are hypotheses that (1) adding a probiotic strain may change the composition of the basic raw material; (2) adding modified buckwheat sprouts to an atherogenic diet may improve the lipid profile.

The study was undertaken to evaluate the effect of the addition of buckwheat sprouts modified with the addition of *Saccharomyces cerevisiae* var. *boulardii* to an atherogenic diet on the metabolism of sterols, stanols and fatty acids in rats.

## 2. Results

### 2.1. Selected General Nutritional Parameters and Biochemical Parameters

During the experiment, rats were fed the following four diets: basic diet AIN-93M (AIN group), basic diet AIN-93M with the addition of lard (HFD group), basic diet with the addition of lard and lyophilisate of control sprouts (HFDCS group), basic diet with the addition of lard and lyophilisate of modified sprouts (HFDPRS). 

Table 1 shows some general nutritional parameters of the rats on experimental diets. The AIN group (970.31 ± 102.20 g) consumed the most significant amount of the diet during the experiment. The remaining groups had amounts comparable to each other, i.e., 669.06 ± 64.68 g (HFD), 674.88 ± 29.25 g (HFDCS) and 671.74 ± 15.03 g (HFDPRS). There is a greater weight gain in the AIN group. However, the lowest gain was in the HFD group. In the HFDCS and HFDPRS groups, the values of weight gain were similar. 

The FER (food efficiency ratio) value differs between the AIN, HFD, and HFDCS, HFDPRS groups. In the groups with the addition of sprouts, the FER value is statistically higher than in the control groups.

Table 2 shows the effect of feeding rats with a diet with the addition of lyophilisates of sprouts on blood biochemical parameters.

The CRP value differed between the groups and decreased in the HFDCS and HFDPRS groups compared to the HFD diet by 55.9%. Taking the AIN-93M diet as a reference, most parameters were reduced in animals compared to the rats fed high-fat diets.

Non-HDL cholesterol values differed statistically between the groups. A higher value of this index was noticed in rats fed with the HFD diet. However, in the groups of rats fed diets with the addition of sprouts, a decrease in the value of this parameter was noted, i.e., 8.71 ± 3.98 mg/dL (HFDCS) and 7.78 ± 3.00 mg/dL (HFDPRS), respectively, compared to the reference group, i.e., HFD.

**Figure 1:**
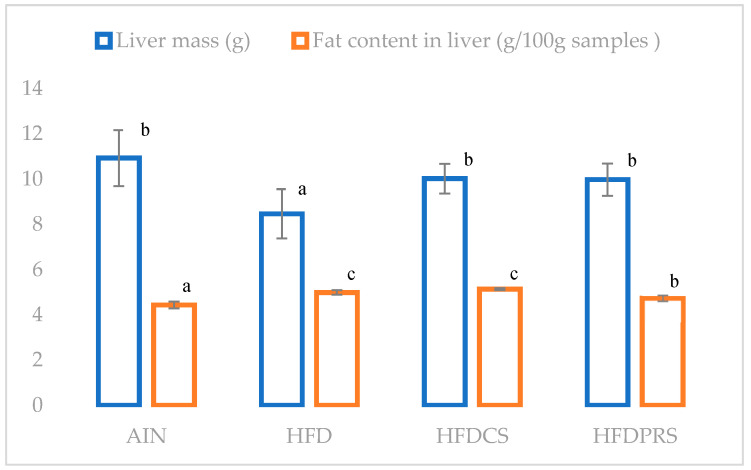
Total fat content and liver mass in experimental groups. Mean values with different letters above the bars differ statistically, *p* < 0.05. Statistical analysis was performed for liver weight and fat content separately. AIN—group fed with the basic diet AIN-93M; HFD—group fed with the basic diet AIN 93M with the addition of lard; HFDCS—group fed with the basic diet AIN-93M with the addition of lard and lyophilisate from control sprouts; HFDPRS—a group fed with the basic diet AIN-93M with the addition of lard and modified buckwheat sprouts lyophilisate.

Figure 1 provides information related to the weight of the liver and the amount of fat in the liver. The high-fat group experienced a reduction in liver weight compared to the rest of the groups. In the HFDCS (5.14 ± 0.04 g/100 g sample) and HFD (4.99 ± 0.10 g/100 g sample) groups, the liver had the highest fat content, followed by HFDPRS (4.74 ± 0.13 g/100 g sample) and AIN (4.44 ± 0.15 g/100 g sample). Statistical analysis was performed for the liver weight and fat content separately.

**Figure 2:**
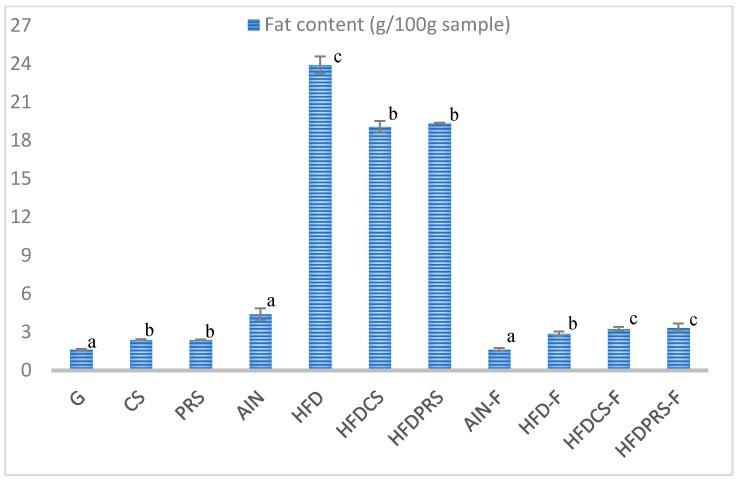
Total fat content in different forms of buckwheat. Mean values with different letters above the bars differ statistically, *p* < 0.05. G—grains, CS—control sprouts, PRS—probiotic-rich sprouts; AIN—group fed with the basic diet AIN-93M; HFD—group fed with the basic diet AIN 93M with the addition of lard; HFDCS—group fed with the basic diet AIN-93M with the addition of lard and lyophilisate from control sprouts; HFDPRS—a group fed with the basic diet AIN-93M with the addition of lard and modified buckwheat sprouts lyophilisate. AIN-F—feces of the AIN group, HFD-F—feces of the HFD group, HFDCS-F—feces of the HFDCS group, HFDPRS-F—feces of the AIN group.

Figure 2 shows the fat content of seeds, sprouts, diets and feces of the animals. The sprouts in the control group (CS) and probiotic-rich sprouts (PRS) had a higher fat content compared to the seeds. In the case of diets, the HFD diet had the highest fat content, followed by the HFDPRS, HFDCS and AIN diets, respectively. In the case of feces, the HFDPRS-F group had the highest fat content, followed by HFCS-F, HFD-F, and AIN-F, respectively.

### 2.2. Sterols

The content of sterols is presented in Table 3. The indicated compounds were identified in seeds, sprouts, diets, livers, feces and serum. A detailed description of the results is presented in the following Section 2.2.1, Section 2.2.2, Section 2.2.3, Section 2.2.4 and Section 2.2.5.

#### 2.2.1. *F. esculentum* Freeze-Dried Grains and Sprouts

Twelve compounds were identified in seeds and sprouts (brassicasterol, campesterol, campestanol, stigmasterol, β-sitosterol, sitostanol, Δ5-avenasterol, α-amyrin, 5,24-stigmastadienol, Δ7-stigmasterol, cycloartanol, 24-methylenecycloartanol). Seeds and control sprouts as well as probiotic-rich sprouts were characterized by the highest content of β-sitosterol, followed by Δ5-avenasterol and campesterol. The highest sterol content characterized the seeds, followed by control sprouts < probiotic-rich sprouts.

#### 2.2.2. Experimental Diets

Eleven compounds were provided with the diet. The highest amount of sterols was provided by the diet AIN (2102.4 ± 12.74 µg/g fat), then HFDPRS (394.64 ± 0.59 µg/g fat), HFDCS (246.42 ± 5.57 µg/g fat), HFD (97.86 ± 0.97 µg/g fat). 

#### 2.2.3. Serum

Cholesterol was identified in the blood serum. The highest amount in 100 µL of serum was found in the AIN-S group, and then in the HFD-S, HFDPRS-S and HFDCS-S groups, respectively. It was noticed that the group fed the diet with the addition of modified buckwheat sprouts had 48.1% lower cholesterol content, compared to the rats fed the AIN diet. 

Sitostanol was also identified in the AIN-S, HFDCS-S and HFDPRS-S groups, with the highest amount in the AIN-S group.

#### 2.2.4. Liver

Seven compounds were identified in the livers. Moreover, in the total content of liver sterols, cholesterol had the highest content. 

Cholesterol content decreased, respectively, in the following groups: HFD-L (32.32 ± 2.76 mg/g fat), HFDCS-L (31.20 ± 3.14 mg/g fat), AIN-L (28.44 ± 2.04 mg/ g fat), HFDPRS-L (24.68 ± 1.96 mg/g fat). It should be noted that the group of rats fed with the diet containing freeze-dried modified sprouts had a significantly lower content of this compound compared to the groups of HFD-L (by 23.6%) and HFDCS-L (by 20.9%).

#### 2.2.5. Feces

Thirteen compounds were identified in the feces, of which campestanol, stigmasterol, α-amyrin, 5,24-stigmastadienol were identified only in selected groups.

Among the identified sterols, cholesterol had the largest share in the total content of sterols in the AIN-F and HFD-F groups. The highest amount was determined in the feces of the HFD-F group (52.04 ± 1.73 mg/g fat), then AIN-F (15.45 ± 2.25 mg/g fat), HFDCS-F (13.53 ± 0.85 mg/g fat), HFDPRS-F (9.47 ± 0.00 mg/g fat). It was found that the group fed the diet with modified buckwheat sprouts had 81.8% lower fecal cholesterol content compared to the rats fed the high-fat diet.

There was a high amount of the following compounds in the stool: brassicasterol, β-sitosterol. The highest content of brassicasterol was determined in feces in the HFDCS-F group, and the lowest in the HFD-F group. In the feces of the HFDPRS-F group (26.98 ± 1.00 mg/g fat), the amount of this compound was lower than in the HFDCS-F group (20.13 ± 0.17 mg/g fat).

The amount of β-sitosterol was identified to be the highest in HFDCS-F (5.77 ± 0.08 mg/g fat) and the lowest in the HFDPRS-F group (4.53 ± 0.07 mg/g fat). There were also statistically significant differences between the groups in the amount of campesterol. The HFDCS-F group had the highest content, followed by HFDPRS-F, HFD-F, AIN-F. The HFDCS-F group had the highest content, followed by HFDPRS-F, HFD-F, AIN-F.

The amount of sitostanol in the stool was the lowest in the HFD-F group, then it increased in the following order: HFDPRS-F (by 19.4%), AIN-F (by 28.2%), and HFDCS-F (by 46.7%).

The HFDPRS-F group was characterized by the lowest content in feces of the following compounds: 5,24-stigmastadienol, 24-methylenecycloartanol, Δ7-stigmasterol, 5,24-stigmastadienol, stigmasterol, cholesterol.

Among the study groups, the HFD-F group had the highest total fecal sterol content. The value was higher than in the HFDPRS-F group by 39.6%.

### 2.3. Fatty Acids

Table 4 shows the identified fatty acids in seeds, sprouts, diets, livers, feces and serum. In contrast, Table 5 lists the amounts of saturated fatty acids (SFA), monoenoic (MUFA) and polyene fatty acids (PUFA). A detailed description of the results is presented in Section 2.3.1, Section 2.2.2, Section 2.3.3 and Section 2.3.4.

#### 2.3.1. *F. esculentum* Freeze-Dried Grains, Sprouts and Experimental Diets

Buckwheat seeds contain a higher content of SFA, MUFA and PUFA, compared to sprouts (Table 5). When comparing the lyophilisate of control sprouts with modified sprouts, it was noticed that the PRS group has a higher content of the indicated groups of fatty acids. Nine fatty acids were identified in seeds, and eleven in control and modified sprouts (Table 4).

As with sprouts, eleven fatty acids have been identified in all diets. Comparing the diets, it was noticed that the HFDCS diet contained the most saturated fatty acids. In the case of monoenoic and polyenic acids, the AIN diet was the highest, followed by the HFDCS, HFDPRS and HFD diets.

Lard, which was part of the diets in the HFD, HFDCS and HFDPRS groups, increased the amount of saturated fatty acids. The lard addition was 200 g/kg of the HFD diet and 200g/1.3 kg of the HFDCS and HFDPRS diets. The differences between the AIN diet and diets with the addition of sprouts were especially noticed in the case of palmitic (C16:0) and stearic (C18:0) acids.

#### 2.3.2. Serum

Among the studied groups, the highest content of saturated fatty acids, monoenic and polyenic fatty acids was found in the AIN-S group (Table 5).

Statistical differences were noticeable when comparing the groups in which sprouts were added to the diets. There is an increase in the total content of monoenoic and polyenic acids in the HFDPRS-S group compared to HFDCS-S (Table 5). The increase was 14.6% and 6.7%, respectively. The following fatty acids C18:1, C20:1, C18:2, C18:3n-3, C18:3n-6, C20:4n-6, C22:5, C22:6 have a significant influence on the differences (Table 4).

#### 2.3.3. Liver

Ten fatty acids were identified in the livers of rats, of which the following acids were not identified in the rats in the HFD-L group: eicosenoic acid (C20:1) and α -linolenic acid (C18:3n-3) (Table 4). 

The livers of rats from all groups had a high content of saturated fatty acids. In the case of PUFA, there is a slight increase in the amount in the HFDPRS-L and HFDCS-L groups compared to the AIN-L and HFD L groups. It was influenced by linoleic acid (C18:2) and γ-linolenic acid. (18:3n-6).

#### 2.3.4. Feces

Ten fatty acids (HFDCS-F groups, HFDPRS-F groups) were identified in the rats’ feces in the experiment. The following acids were not detected in the HFD-F group: arachidic (C20:0); docosanoic (C22:0). However, in the AIN-F group, no acids were detected, i.e., arachidic (C20:0), docosanoic (C 22:0); palmitolenic acid (C16:1).

The amount of excreted polyenic acids was higher in the AIN-F group by 61.5%, compared to the HFDPRS-F group. In total, the amount of saturated fatty acids in the stool was lower in the HFD-F, HFDCS-F, and HFDPRS-F groups, compared to the liver’s content.

## 3. Discussion

### 3.1. Sterols

Plant sterols and stanols are effective in lowering serum LDL cholesterol. Hence, they may play an important role in the development of atherosclerotic lesions. Both sterols and plant stanols are useful for hypercholesterolemic patients. They can be in addition to cholesterol-lowering drugs or to the diet. Reduced intestinal cholesterol absorption leads to increased cholesterol synthesis and increased expression of the LDL receptor. The effect on VLDL production is not known, and it is unclear whether bile formation and/or composition has changed. Serum triacylglycerol and HDL-cholesterol levels were unchanged in most studies. Various suggested mechanisms can be found in the literature to explain the mechanisms behind the cholesterol-lowering effects of plant sterols and stanols. Plant sterols can displace cholesterol from mixed micelles. This is because they are more hydrophobic than cholesterol. Such a replacement reduces the concentration of micellar cholesterol. As a consequence, cholesterol absorption is lowered. It should also be noted here that plant sterols or stanols can reduce the rate of cholesterol esterification in enterocytes, and consequently the amount of cholesterol excreted via chylomicrons [2,17,18].

The review by Raguindin et al. in 2020 showed that the total content of phytosterols ranged from 35 mg/100 g to 68.2 mg/100 g in oats, while in buckwheat from 19 mg/100 g to 139 mg/100 g. The most abundant sterol in buckwheat and oats was sitosterol, constituting over 50–70% of all sterols, followed by campesterol [19]. In this study, the highest amount of sitostanol in the stool was found. However, in sprouts, diets, liver, and serum, they are significantly lower.

In seeds, control sprouts and probiotic-rich sprouts, 20.81 ± 1.93 mg/g lipids (33.71 mg/100 g product), 20.85 ± 1.88 mg/g lipids (50.46 mg/g product), 19.00 ± 2.18 mg/g lipids (45.79 mg/100 g of the product) were recorded, respectively. On the other hand, in the case of the tested material, both in seeds and sprouts, β-sitosterol was found in the highest amounts among the remaining compounds.

In a study by Yang et al in 2014, it was shown that in Tartary buckwheat, the following sterols, β-sitosterol, campesterol, had the largest share in the total amount of sterols [20]. 

It was noticed that the addition of the sprout lyophilisate to the atherogenic diet increased the total content of sterols in HFDCS diets (246.42 ± 5.57 µg/g lipids) and HFDPRS (394.64 ± 0.59 µg/g lipids) compared to the HFD diet (97.86 ± 0.97 µg/g lipids). There are no significant differences between the following HFD-S, HFDCS-S, HFDPRS-S groups in the total sterol content. By comparing the biochemical parameters, it can be observed that the HFDCS and HFDPRS groups had a significantly lower content of LDL-C cholesterol in the blood serum compared to the HFD group.

In a study by Liu et al in 2021, male Sprague Dawley rats were fed a high-fat diet for 6 weeks. They were then orally dosed with buckwheat protein (TBP) over a five-week period. The study showed that in serum, TBP supplement significantly lowered LDL-C levels and raised HDL-C levels [21].

*Saccharomyces crevisiae* var. *boulardii* may have the ability to alter cholesterol levels. The mechanism indicated by the researchers is the assimilation of cholesterol. It was shown in a study by Ryan et al. in 2015 that *Saccharomyces boulardii* removed cholesterol from laboratory culture media. This was carried out through its assimilation in yeast cells. Accordingly, *Saccharomyces cerevisiae* var. *boulardii* can assimilate intestinal cholesterol and subsequently alter serum cholesterol levels [22]. In the analyzed study, it can be noticed that rats fed a diet with the addition of control sprouts had a lower serum cholesterol content compared to the group fed an atherogenic diet with probiotic-rich sprouts. 

Plant sterols are poorly absorbed in the intestine (0.4–3.5%), while plant stanols (0.02–0.3%) are absorbed even less. However, it is worth noting for comparison that the absorption of cholesterol ranges from 35 to 70%. There may be gender differences in this case. This is because the absorption of plant sterols and stanols in female rats appears to be higher than in male rats. Plant stanols can also reduce the absorption of plant sterols, and vice versa [2]. One of the reasons for the low absorption of plant stanols and sterols can mean plant sterols and stanols are weak esterified [23].

There is an indication in the works that the extent and rate of absorption of plant sterol or stanol depends on the length of the side chain and the presence of the five double bonds (saturation). Other factors may alter the intestinal absorption of sterols or stanols. Such factors may be mutations and polymorphisms in the ABCG5 or ABCG8 gene. An example is sitosterolemia, which is a rare autosomal recessive disease. It causes the accumulation of plant sterols and stanols and can lead to severe atherosclerosis at a very young age [2,24].

The enterohepatic metabolism of cholesterol and plant sterols is complex. In the lumen of the intestine, dietary cholesterol (and plant sterols) is reduced to free sterols by esterases and transferred to the micelles. Micelles are a mixture of bile salts, phospholipids, free sterols and some fatty acids. They interact with the apical membrane of enterocytes (a process that has not been characterized at the molecular level). They allow sterols to enter enterocytes. Cholesterol is esterified with ACAT-2 and then incorporated into chylomicrons, which in turn are secreted at the basolateral surface into the lactealss. Eventually, they drain into the venous circulation. Lipoprotein lipase acts on the chylomicrons in the capillary beds of all organs. This allows the body to supply these tissues with triglycerides from the diet and some fat-soluble vitamins. The sterols in these molecules are not transferred and remain part of the remaining molecules. The particles are now recognized by the receptors in the liver and then purified. As a result, most of the sterols are delivered to the liver. Cholesterol enters the metabolic pool and can be repackaged into VLDL and excreted back into the circulation. When non-cholesterol sterols are excreted into the bile ducts, they return back to the intestinal lumen [25].

Unabsorbed cholesterol and plant stanols are excreted in the feces. Decreasing cholesterol absorption may increase its synthesis in the liver, and these amounts do not compensate for the losses; therefore, the level of total cholesterol and LDL fraction is lowered in the blood serum [3].

### 3.2. Fatty Acids

N-6 fatty acids, including linoleic acid (e.g., found in plant seed oils), are known to lower serum cholesterol, so it is generally recommended to partially replace dietary SFA with n-6 acids. With regard to PUFA, such dietary changes have been a hallmark of clinical trials. It is worth noting that PUFA-rich diets inhibit high-density lipoprotein (HDL), which protects against CHD, and also lowers LDL [26].

Diseases such as hyperlipidemia or atherosclerosis can be caused by a person’s excessive consumption of high-fat and sugar products. This can happen by disrupting the lipid metabolism. Serum TG, TC, LDL-C, and HDL-C levels are major indicators that may reflect the body’s lipid metabolism. An abnormal increase in blood lipids may lead to disease-threatening conditions [21].

In the study by Ðurendic-Brenesel et al. in 2013, it was shown that supplementation with a mixture of buckwheat leaves and flowers significantly reduced body weight gain, plasma lipid concentration and atherogenic index in rats fed a high-fat diet [27].

In the publication by Raguindin et al. in 2021, it was indicated that the fatty acid content of oats and buckwheat was mainly composed of high levels of unsaturated fatty acids, i.e., oleic (C18:1) and linoleic (C18:2) acids; and palmitic acids (C16:0), stearic acid (C18:0) and linoleic acid (C18:3). Changes in the relative abundance of fatty acids with different anatomical parts of the plant; the effects of various environmental growth factors and genetic variants have been noted [19]. In the presented study, the highest amounts of linoleic acids (C18:2), unsaturated oleic (C18:1) and saturated palmitic (C16:0) acids were identified in both sprouts and seeds. Modification of seeds by adding a probiotic yeast strain influenced the content of the indicated acids in sprouts (it is higher in sprouts rich in probiotics than in control sprouts).

It was demonstrated in the study by Ðurendic-Brenesel et al. in 2013 that a group of animals fed a high-fat diet with the addition of buckwheat leaves and flowers significantly increased the content of n-6 fatty acids and eicosapentaenoic acid (EPA). However, it reduced the content of saturated fatty acids (SFA) and oleic acid [27].

In the literature, there is a concept related to lipids of microbial origin, which are referred to as single cell oil (SCO). They are produced by microorganisms known as oleaginous microorganisms. They include molds, microalgae, bacteria and yeasts. They are defined as organisms capable of “producing and accumulating more than 20% of dry cell substance” [28,29,30]. Molska et al. in 2020 indicate that although *Saccharomyces cerevisiae* has not yet been identified in the literature as an oleaginous microorganism, this species is more often referred to as non-oil yeast. However, there are studies showing their effect on fatty acid production. *De novo* synthesis mechanisms stand out here, which consists of obtaining fat from acetyl-CoA and malonyl-CoA molecules [31]. The fatty acids synthesized by oleaginous microorganisms are primarily palmitic, stearic, myristic oleic, linoleic, linolenic and palmitoleic acids [28,29]. In this work, this mainly applies to linoleic and linolenic acids, which, when comparing the control with probiotic-rich sprouts, shows an increase in its amount. In the experiment, the AIN-93M diet and atherogenic diets with the addition of common buckwheat sprouts were used. An increase in the amount of monoenoic and polyoic acids was observed in the HFDPRS group compared to the HFD group. Reduction in saturated fatty acids was also observed, compared to the HFDCS group.

The liver is the central organ, controlling lipid homeostasis through complex but precisely regulated signaling, biochemical and cellular pathways. Liver cells, the so-called hepatocytes, are the main parenchymal cells of the liver. They control metabolic functions in the liver, including triglyceride metabolism and biochemical functions. Fatty acids accumulate in the liver through de novo biosynthesis and uptake of liver cells from plasma. They are eliminated either by oxidation in the cell or by secretion into the plasma in triglyceride-rich very low density lipoproteins [32]. There were no significant differences between the atherogenic diet and the atherogenic diet with the addition of sprouts in blood biochemical parameters. Compared to the AIN control diet, a significant decrease in this parameter was observed.

In the livers of the HFDPRS-L group, a higher amount of C18: 0, C23: 0, C18: 3n-6 acids was observed, compared to the group fed with the control sprouts. In the serum, comparing the indicated groups, a greater amount of the following acids was found in the HFDPRS group: C16:0, C18:0, C18:1, C20:1, C18:2, C18:3n-3, C18:3n-6, C20:4n-6, C22:5, C22:6.

The indicated data coincide with the reports of other authors [19,23,32,33,34]. 

In the serum, when comparing the HFD-S, HFDCS-S and HFDPRS-S groups, a greater total amount of monoenoic and polyenoic acids was observed compared to the HFDCS group.

Dietary fatty acids are built into tissues and blood, and the fatty acid composition of these tissues is often used as biomarkers of fat intake. It is worth mentioning that both the amount and composition of fecal fatty acids reflect fat intake, intestinal fatty acid absorption and activity of colon bacteria [35].

In the faeces of animals from the HFDPRS-F group, a greater amount of excreted saturated fatty acids was found than in the HFD-F group.On the other hand, a lower amount of monoenoic and polyenic acids was observed, which may indicate a higher use of them by the body.

## 4. Materials and Methods

### 4.1. Animals

Thirty-two male Wistar albino rats (~8 weeks old) were used in the study. All the experimental procedures were approved by the local bioethics committee for animal studies (approval number 28/2017). Rats were housed on a 12-h light/dark cycle, thermostatically (20 °C ± 2) and with 55–65% humidity throughout the adaptation and experiment period. The mean weight of the rats was 188.7 ± 14.1 g. The experiment lasted 6 weeks.

#### 4.1.1. Experimental Diets

The composition of the diets is presented in Table 6. The diets were developed on the basis of the AIN-93M diet modification [36]. Semi-synthetic diets consisted of wheat starch (Celiko, Poznań, Poland), potato starch (on potatoes from Iława, Poland), casein (from Murowana Goślina, Poland), soybean oil (ZPT Warsaw, Poland), sucrose (Diamant, Pfeifer & Langen Polska S.A., Poznań, Poland), choline (Sigma-Aldrich, Darmstadt, Germany), mineral mix (AIN-93M-MX) [36], vitamin mix (AIN-93-VX) [36] and choline (Sigma-Aldrich). In addition, the sprouts were added in the form of a lyophilisate to the HFDCS and HFDPRS diets. The diets were prepared by mixing all the ingredients.

##### Buckwheat Seeds

Buckwheat seeds (*Fagopyrum esculentun* Moench) were purchased from PNOS S.A. in Ożarów Mazowiecki. The strain of *Saccharomyces cerevisiae* var. *boulardii* was grown for 48 h at 30 °C on malt agar. Then, the colonies were sterile picked and suspended in water. First, the seeds were disinfected with 1% (*v*/*v*) sodium hypochlorite (Sigma-Aldrich, St. Louis, MO, USA) for 10 min. They were then filtered and washed with distilled water until the pH was neutral. After reaching the expected pH, they were placed in an aqueous suspension of *Saccharomyces cerevisiae* var. *boulardii*, at a level of 1 × 10^7^ mL^−1^ based on the OD value (probiotic-rich sprouts) or in distilled water for 4 h (control sprouts). The seeds were then placed in a growth chamber (SANYO MLR 350H) on Petri dishes (ϕ 125 mm) lined with absorbent paper, where they germinated in the dark for three days. Sprouting was run at 30 °C. After three days, sprouts were manually collected and rinsed with distilled water [31].

#### 4.1.2. Experimental Design

Before starting the experiment, the animals were adapted to laboratory conditions. It lasted three days. During this period, animals had unlimited access to water and standard AIN-93M diet [36]. The animals were kept in stainless steel cages covered with non-metal glaze (throughout the adaptation and experimentation period).

After this period, the rats were randomized into four groups, each consisting of eight individuals. One of the groups was fed the AIN-93M diet; the next three groups were fed with the modified AIN-93M diet with the addition of lard in the amount of 200 g/kg for the HFD diet and 200 g/1.3 kg for the HFDCS and HFDPRS diets (Table 6) [36]. The addition of 600 g of sprouts, control and modified sprout lyophilisate, respectively, was included in the HFDCS and HFDPRS diets.

Each day, the animals were given a fresh portion of food and water, and any food and water residues from the previous day were removed. Diet and water consumption was monitored daily and the body weight of the rats was monitored weekly. The digestibility of the rats was determined during the experiment.

At the end of the experiment, the rats were weighed and then euthanized using carbon dioxide inhalation. During the section, the internal organs, including liver in this range, were removed, washed in saline, weighed and stored at −80 °C. Blood samples were collected after 12 h fast by cardiac puncture in serum-separated tubes to obtain serum.

#### 4.1.3. Biochemical Parameters

Blood was collected by cardiac puncture into sodium heparin tubes to obtain whole blood for complete blood counts, and into tubes separated from the serum for the biochemical parameters. The coagulated blood was allowed to clot at room temperature for 30 min and then centrifuged for 15 min at 3600× *g*. The following biochemical parameters were determined: glucose (GLU), triacylglycerols (TAG), activity of alanine transaminase (ALA), activity of aspartate transaminase (AST), total cholesterol (TCH), high-density lipoprotein cholesterol (HDL), non-HDL and protein C -reactive (CRP). Morphological index values were determined using a Sysmex K-1000 hematology analyzer (TAO Medical Electronics Co., Kobe, Japan) according to standard procedures. Serum glucose concentration was estimated by the glucose oxidase method. Serum total cholesterol and triglyceride levels were measured using commercial kits (Randox Laboratory Ltd., Crumlin, UK). The activity of liver enzymes, such as ALT and AST, was determined according to Dembińska-Kiec and Nastalski [37].

### 4.2. Determination of Crude Fats

Fat content in lyophilisates, seeds, controls and modified sprouts; liver, experimental diets and feces were determined using the Avanti Soxtec system (Model 2055 Manual Extraction Unit; Foss Tecator, Höganäs, Sweden), according to the AOAC Official Method 945.16 [38].

### 4.3. Fat Extraction

#### 4.3.1. Fat Extraction from Freeze-Dried Seeds; the Freeze-Dried Control Sprouts; Freeze-Dried Sprouts Rich in Probiotics; Experimental Diet; Feces

Extraction of fat from freeze-dried seeds, freeze-dried control sprouts, probiotic-rich freeze-dried sprouts, experimental diet and feces were extracted according to the procedure described by Folch et al. (1957) [39]. The amount of fat was quantified and expressed as mg/g lipids (in buckwheat seeds and sprouts; liver; feces), µg/g lipids (in experimental diets) and µg/100 µL serum (in serum).

#### 4.3.2. Hepatic-Fat Extraction

Three times ~1 g of tissue was collected from the scattered zones of the frozen left flap. The exact wet weight of each sample was determined after thawing and dehydration of excess moisture on the filter paper for 10 min at 25 °C. Total fat was extracted from liver samples using the Folch method with a slight modification [39,40]. Each sample (~1 g of tissue) was mechanically homogenized in 25 mL of chloroform-methanol (2:1) solution for 2 min. The homogenate was sonicated (VCX 750, Sonics and Materials Inc., Newtown, CT, USA) for 5 min at amplitude of 30%, with 5 s cycles on, 5 s off. The sonicated samples were then shaken overnight (12 h) at 25 °C. The wet mass from the solution was separated into a volumetric flask on the filter. The same amount of distilled water was then added as the amount of liquid in the flask. Sodium sulfate was placed on the filter to remove traces of residual water, and the contents were poured into a test tube. After separation of the layers, the layer of choloroform was transferred to another weighed test tube, the content of which was evaporated to dryness under liquid nitrogen. Then, the tubes were weighed to determine the amount of fat. The amount of fat was quantified.

#### 4.3.3. Serum

The serum was stored at −80 °C. Fatty acids were extracted with the modified Folch method [39,41], briefly Folch reagent (2:1 chloroform:methanol), for lipid extraction from cells; butylated hydroxytoluene as an antioxidant and internal standard: deuterated myristic acid-d_27_ (d_27_C14:0) in chloroform was added to the weighed samples and centrifuged. Extraction of the fat for the determination of sterols was performed in the same manner, except that the internal standard 5α-cholestane was added to the samples instead of the above-mentioned standard.

### 4.4. Analysis of the Fatty Acid Composition of: Freeze-Dried Seeds, Freeze-Dried Control Sprouts, Lyophilized Sprouts Rich in Probiotics, Experimental Diet, Liver, Feces

For the determination of fatty acids, they were converted into fatty acid methyl esters according to the method of AOCS Official Method Ce 2b-11 [42].

The heptadecanoic acid ester (0.25 mg/sample) was used as an internal standard. The samples were saponified with a 1 M solution of KOH in methanol. Then, they were incubated in a heating block for 20 min at 70 °C. The samples were cooled down and only then 5 mL of a 14% solution of BF3 in methanol was added to them. Again, the samples were incubated at 70 °C, for 20 min. After cooling, 2.5 mL of hexane was added to the samples. After mixing, 2.5 mL of water was added. After phase separation, 1 µL of hexane layer was taken and subjected to statistical analysis.

The gas chromatograph was a Trace 1300 with FID detector (Thermo Scientific, Waltham, MA, USA), while a SP TM-2560 capillary column (100 m × 0.25 mm × 0.2 µm) (Supelco, Bellefonte, PA, USA) was used for the analysis. The carrier gas was hydrogen (1.5 mL/min). The analysis was performed in splitless mode. The initial oven temperature was 160 °C for 1 min, and then it was increased 6 °C/min to 220 °C and it remained at this temperature for 17 min. The inlet and detector temperature were 240 °C. The retention times were compared to the retention times of 37 Component Fame Mix (Supelco, USA).

### 4.5. Analysis of Fatty Acid Composition of Serum 

Approximately 2 mL of the lipid-containing chloroform phase was removed and derivatized with 0.5 mL of 0.5 M potassium hydroxide in methanol and 1 mL of boron trifluoride methylation by heating at 70 °C. Fatty acid methyl esters (FAME) were extracted into 4 mL of hexane and washed with 2 mL of distilled water. The hexane (organic) phase was then transferred to 2 mL vials. Then, the samples were analyzed by gas chromatography. The aforementioned analysis was carried out on a Hewlett-Packard 6890 gas chromatograph (Wilmington, DE, USA) equipped with a split/splitless injector and a flame ionization detector (FID). FAMEs were separated using a SelectFame column (50 m × 0.25 mm × 0.25 μm, Agilent Technologies, Santa Clara, CA, USA), identified by comparison with the available FAME standards (Supelco, Bellefonte, PA, USA). Fatty acid content was calculated by comparing the area of individual peaks with the area of the peak of the internal standard and recalculated on the basis of the sample weight Response factors (FID) for individual fatty acids compared to internal standards were taken as unity. The concentration of fatty acids was expressed quantitatively [41].

### 4.6. Analysis of the Composition of Sterols: Freeze-Dried Seeds, Freeze-Dried Control Sprouts, Lyophilized Sprouts Rich in Probiotics, Experimental Diet, Liver, Serum, Feces

The content of sterols and stanols was determined according to the AOCS Official Method Ch 6-91 [43]. For determinations, 50 mg of fat was taken and 50 µg of the internal standard 5α-cholestane was added, which was saponified with 1 M KOH in methanol. Extraction of the sterol fraction was performed using the hexane: MTBE system (1:1, *v*/*v*). The solvent was evaporated under nitrogen. After evaporation of the solvent, the residue was dissolved in 100 µL of pyridine and silylated with BSTFA reagent with 1 µ TMCS. The phytosterols were analyzed using a Hewlett-Packard 6890 gas chromatograph in splitless mode with an FID detector. A DB-35MS 30 m × 0.25 mm × 0.25 µm capillary column was used (J&W Scientific, Folsom, CA, USA). The injector and detector were kept at 300 °C; the oven temperature was initially 100 °C for 5 min, increasing at 25 °C/min to 250 °C and then at 3 °C/min to 290 °C. The final temperature was held for 20 min. The carrier gas was hydrogen and the flow rate was 1.5 mL/min. Sterols were identified by comparing their retention times with those of standards. The sterols were determined in duplicate. The concentration of fatty acids was expressed quantitatively.

### 4.7. Statistical Analysis

Statistical analysis of the data was performed using Statistica 10 (StatSoft, Tulsa, OK, USA). For statistical analysis, one-way analysis of variance and intergroup differences was used by Tukey’s HSD post-hoc test with a significance level of *p* < 0.05. Significant differences were denoted with different superscript letters.

## 5. Conclusions

The study attempts to evaluate the effect of the addition of buckwheat sprouts modified with the addition of *Saccharomyces cerevisiae* var. *boulardii* to an atherogenic diet on the metabolism of sterols, stanols and fatty acids in rats. The analysis began with the analysis of the raw material, and then the diet, nutritional and biochemical parameters, as well as liver, serum and feces.

Based on the results, changes in the composition as well as the amounts of individual sterols, stanols and fatty acids were noticed. The addition of probiotic-rich sprouts with lyophilisate to the diet increased the amount of sterols compared to other atherogenic diets. There were 75.2% more sterols in the HFDPRS diet compared to the HFD diet.

It was noted that the group in which the modified sprouts were added to the diet had both a lower amount of cholesterol in the liver and feces compared to the rats fed a high-fat diet. The values are lower by 23.6% and 81.8%, respectively.

In the case of fatty acids, changes can be observed when comparing CS and PRS sprouts, including a reduction in the amount of saturated fatty acids in the group of modified sprouts, as well as a 35.5% increase in the amount of polyenic acids, which is of particular importance in the aspect of cardiovascular diseases.

Significant changes are observed in the range of fatty acids in the serum, liver, feces. There are statistically significant differences in the content of individual monoenoic and polyenic acids in the serum of rats fed the HFDPRS diet, compared to rats fed the HFDCS diet. It is influenced, among others, by acids such as C18:2, C20:4n-6, C22:6. They increase compared to the HFDCS group by 13.5%, 15.1% and 13.1%, respectively.

In the case of the HFDPRS group, there is a noticeably lower excretion of monoenoic and polyenic acids compared to the other studied groups. It is also worth mentioning here that the addition of sprouts to the atherogenic diet lowered the CRP index, i.e., acute phase proteins.

Referring to the hypotheses in the work, the following points can be made:

(1) It can be observed that the modification changed the content of individual compounds contained in the raw material, i.e., buckwheat. This is especially noticeable for the fatty acids C18:1, C18:2, C18:3n-3;
(2) Changes were noticed in the lipid profile by comparing the HFD and HFDPRS groups. In the HFDPRS group, a lower value of the non-HDL and LDL-C index was noticed.

It is advisable to conduct further research related to the metabolism of both sterols, stanols and fatty acids in the body in terms of the use of probiotic microorganisms. As it can be concluded from the obtained results, they indicate that the addition to the diet of the modified *F. esculentum* buckwheat seeds by the addition of the probiotic strain of the yeast *S. cerevisiae* may have a significant impact on the metabolism of the indicated components in the body.

The current trend of searching for new functional raw materials offers many opportunities for both technologists and dieticians. The presented research provides a new direction for the use of the raw material, buckwheat sprouts.

## Figures and Tables

**Table 1:** Selected general nutritional parameters of rats on experimental diets.

Groups	*n*	Parameters			
		Consumed Diet (g)	FER *	Initial Weight (g)	Weight Gain (g)
AIN	8	970.31 ± 102.20 ^b^	0.20 ± 0.02 ^a^	188.88 ± 23.71 ^a^	190.00 ± 26.27 ^b^
HFD	8	669.06 ± 64.68 ^a^	0.20 ± 0.03 ^a^	191.63 ± 12.21 ^a^	134.88 ± 24.47 ^a^
HFDCS	8	674.88 ± 29.25 ^a^	0.25 ± 0.02 ^b^	187.50 ± 14.36 ^a^	171.50 ± 9.68 ^b^
HFDPRS	8	671.74 ± 15.03 ^a^	0.26 ± 0.02 ^b^	187.13 ± 15.09 ^a^	176.75 ± 14.27 ^b^

* FER (food efficiency ratio) = weight gain (g)/food intake (g) × 100. AIN—group fed with the basic diet AIN-93M; HFD—group fed with the basic diet AIN 93M with the addition of lard; HFDCS—group fed with the basic diet AIN-93M with the addition of lard and lyophilisate from control sprouts; HFDPRS—a group fed with the basic diet AIN-93M with the addition of lard and modified buckwheat sprouts lyophilisate. The mean values with different letters in the row are statistically different (*p* < 0.05). “±” indicates standard deviation.

**Table 2:** Influence of the diets with or without probiotic-rich sprouts on biochemical parameters in rats.

Parameters	AIN	HFD	HFDCS	HFDPRS
ALT (U/L)	26.30 ± 7.68 ^a^	26.24 ± 10.38 ^a^	24.74 ± 8.09 ^a^	24.54 ± 4.27 ^a^
AST (U/L)	104.73 ± 24.27 ^a^	101.54 ± 56.28 ^a^	96.56 ± 23.58 ^a^	117.91 ± 45.75 ^a^
TCH (mg/dL)	66.86 ± 3.13 ^a^	79.25 ± 10.86 ^b^	70.35 ± 6.94 ^a^	70.19 ± 8.10 ^ab^
HDL-C (mg/dL)	60.30 ± 2.83 ^a^	64.74 ± 8.37 ^a^	61.64 ± 5.83 ^a^	62.41 ± 7.79 ^a^
Non-HDL (mg/dL)	7.17 ± 1.46 ^a^	14.51 ± 5.57 ^b^	8.71 ± 3.98 ^a^	7.78 ± 3.00 ^a^
LDL-C (mg/dL)	7.29 ± 1.86 ^a^	16.13 ± 4.27 ^b^	11.14 ± 2.88 ^a^	10.04 ± 2.26 ^a^
CRP (mg/L)	0.31 ± 0.03 ^a^	0.68 ± 0.39 ^b^	0.30 ± 0.01 ^a^	0.30 ± 0.01 ^a^
GLU (mg/dL)	7.41 ± 2.25 ^ab^	4.68 ± 2.29 ^a^	6.85 ± 2.10 ^ab^	8.21 ± 2.28 ^b^
TAG (mg/dL)	96.49 ± 19.36 ^b^	53.69 ± 15.51 ^a^	63.54 ± 17.26 ^a^	66.54 ± 8.57 ^a^

The mean values with different letters in the row are statistically different (*p* < 0.05). “±” indicates standard deviation. Each group consisted of (*n*) eight rats. AIN—group fed with the basic diet AIN-93M; HFD—group fed with the basic diet AIN 93M with the addition of lard; HFDCS—group fed with the basic diet AIN-93M with the addition of lard and lyophilisate from control sprouts; HFDPRS—a group fed with the basic diet AIN-93M with the addition of lard and modified buckwheat sprouts lyophilisate.

**Table 3:** The content of sterols in buckwheat seeds (mg/g lipids) and sprouts (mg/g lipids), experimental diets (µg/g lipids), serum (µg/100 µL serum), liver (mg/g lipids) and feces (mg/g lipids).

Sample	Cholesterol	Brassicasterol	Campesterol	Campestanol	Stigmasterol	β-Sitosterol	Sitostanol	Δ5-Avenasterol	α-Amryin	5,24-Stigmasta-Dienol	Δ7-Stigmasterol	Cycloartanol	24-Methylene-cycloartanol	Total Sterols
G	ND	0.30 ± 0.09 ^a^	1.85 ± 0.42 ^a^	0.14 ± 0.03 ^a^	0.35 ± 0.05 ^a^	13.7 ± 1.80 ^a^	0.40 ± 0.06 ^a^	2.51 ± 0.19 ^b^	0.42 ± 0.09 ^a^	0.14 ± 0.02 ^a^	0.34 ± 0.09 ^a^	0.20 ± 0.03 ^a^	0.34 ± 0.03 ^a^	20.81 ± 1.93 ^a^
CS	ND	0.30 ± 0.08 ^a^	1.97 ± 0.47 ^a^	0.17 ± 0.04 ^a^	1.52 ± 0.28 ^b^	12.8 ± 1.24 ^a^	0.40 ± 0.09 ^a^	2.31 ± 0.30 ^ab^	0.35 ± 0.17 ^a^	0.14 ± 0.06 ^a^	0.41 ± 0.08 ^a^	0.16 ± 0.03 ^a^	0.26 ± 0.05 ^a^	20.85 ± 1.88 ^a^
PRS	ND	0.31 ± 0.08 ^a^	1.69 ± 0.36 ^a^	0.16 ± 0.04 ^a^	1.48 ± 0.26 ^b^	11.67 ± 1.47 ^a^	0.38 ± 0.07 ^a^	2.00 ± 0.36 ^a^	0.31 ± 0.11 ^a^	0.18 ± 0.05 ^a^	0.32 ± 0.07 ^a^	0.20 ± 0.07 ^a^	0.30 ± 0.09 ^a^	19.00 ± 2.18 ^a^
AIN	ND	76.96 ± 4.52 ^a^	459.43 ± 0.81 ^d^	16.21 ± 0.11 ^a^	101.03 ± 0.42 ^d^	1093.56 ± 9.11 ^d^	70.09 ± 0.12 ^d^	44.89 ± 3.30 ^c^	ND	93.11 ± 1.97 ^c^	57.06 ± 1.51 ^c^	25.20 ± 0.11 ^c^	64.92 ± 4.79 ^c^	2102.40 ± 12.74 ^d^
HFD	ND	ND	38.46 ± 1.82 ^a^	ND	4.37 ± 0.23 ^a^	35.10 ± 1.23 ^a^	2.19 ± 0.05 ^a,^	2.28 ± 0.18 ^a^	ND	3.30 ± 0.21 ^a^	2.78 ± 0.07 ^a^	5.11 ± 0.42 ^a^	4.29 ± 0.35 ^a^	97.86 ± 0.97 ^a^
HFDCS	ND	ND	65.94 ± 3.26 ^b^	ND	10.48 ± 0.74 ^b^	108.99 ± 1.44 ^b^	4.12 ± 0.17 ^b^	14.71 ± 0.48 ^b^	ND	7.43 ± 0.05 ^b^	8.85 ± 0.14 ^b^	18.92 ± 0.51 ^b^	6.99 ± 0.49 ^a^	246.42 ± 5.57 ^b^
HFDPRS	ND	ND	96.68 ± 5.63 ^c^	ND	16.06 ± 0.11 ^c^	179.40 ± 3.01 ^c^	9.43 ± 0.32 ^c^	20.71 ± 0.38 ^b^	ND	8.76 ± 0.04 ^b^	10.75 ± 0.04 ^b^	30.96 ± 0.64 ^d^	21.90 ± 1.56 ^b^	394.64 ± 0.59 ^c^
AIN-S	49.00 ± 1.31 ^b^	ND	ND	ND	ND	ND	1.23 ± 0.18 ^b^	ND	ND	ND	ND	ND	ND	47.78 ± 3.06 ^b^
HFD-S	26.58 ± 1.04 ^a^	ND	ND	ND	ND	ND	ND	ND	ND	ND	ND	ND	ND	26.58 ± 3.58 ^a^
HFDCS-S	19.38 ± 1.10 ^a^	ND	ND	ND	ND	ND	0.64 ± 0.05 ^a^	ND	ND	ND	ND	ND	ND	20.03 ± 5.43 ^a^
HFDPRS-S	25.45 ± 0.52 ^a^	ND	ND	ND	ND	ND	0.67 ± 0.03 ^a^	ND	ND	ND	ND	ND	ND	26.12 ± 3.71 ^a^
AIN-L	28.4 ± 2.04 ^ab^	ND	0.35 ± 0.08 ^b^	0.11 ± 0.00 ^c^	0.07 ± 0.00 ^bc^	0.57 ± 0.05 ^c^	0.01 ± 0.00 ^a,^	0.08 ± 0.00 ^bc^	ND	ND	ND	ND	ND	29.64 ± 2.06 ^ab^
HFD-L	32.32 ± 2,76 ^b^	ND	0.14 ± 0.04 ^a^	0.04 ± 0.00 ^a^	0.09 ± 0.02 ^c^	0.17 ± 0.02 ^a^	ND	0.09 ± 0.01 ^c^	ND	ND	ND	ND	ND	32.84 ± 2.75 ^b^
HFDCS-L	31.20 ± 3.14 ^b^	ND	0.20 ± 0.07 ^a^	0.07 ± 0.01 ^b^	0.04 ± 0.01 ^a^	0.40 ± 0.07 ^b^	0.01 ± 0.00 ^a^	0.05 ± 0.01 ^a^	ND	ND	ND	ND	ND	31.93 ± 3.11 ^b^
HFDPRS-L	24.68 ± 1.96 ^a^	ND	0.13 ± 0.05 ^a,^	0.07 ± 0.01 ^b^	0.05 ± 0.01 ^ab^	0.38 ± 0.03 ^b^	ND	0.07 ± 0.01 ^b^	ND	ND	ND	ND	ND	25.30 ± 1.98 ^a^
AIN-F	15.45 ± 2.25 ^a^	12.81 ± 1.81 ^b^	0.64 ± 0.01 ^a^	ND	ND	5.27 ± 0.37 ^ab^	1.56 ± 0.11 ^b^	0.11 ± 0.00 ^a^	ND	ND	0.97 ± 0.04 ^b,^	0.78 ± 0.08 ^ab^	1.38 ± 0.15 ^b^	38.96 ± 4.64 ^a^
HFD-F	52.04 ± 1.73 ^b^	5.40 ± 0.88 ^a^	2.37 ± 0.11 ^b^	0.83 ± 0.08 ^a^	1.33 ± 0.23 ^c^	5.55 ± 0.07 ^b^	1.12 ± 0.03 ^a^	0.33 ± 0.03 ^b^	ND	1.52 ± 0.02 ^c^	0.62 ± 0.03 ^b^	0.48 ± 0.06 ^a^	1.14 ± 0.22 ^ab^	72.72 ± 3.29 ^b^
HFDCS-F	13.53 ± 0.85 ^a^	26.98 ± 1.00 ^d^	5.16 ± 0.19 ^d^	1.00 ± 0.00 ^b^	0.45 ± 0.01 ^ab^	5.77 ± 0.08 ^b^	2.10 ± 0.14 ^c^	1.97 ± 0.04 ^d^	0.55 ± 0.14 ^a^	1.15 ± 0.04 ^b^	1.15 ± 0.01 ^c^	1.02 ± 0.15 ^b^	1.45 ± 0.11 ^b^	62.27 ± 1.92 ^b^
HFDPRS-F	9.47± 0.00 ^a^	20.13 ± 0.17 ^c^	3.15 ± 0.23 ^c^	ND	0.60 ± 0.04 ^b^	4.53 ± 0.07 ^a^	1.39 ± 0.10 ^ab^	1.35 ± 0.00 ^c^	0.44 ± 0.04 ^a^	0.69 ± 0.02 ^a^	0.65 ± 0.03 ^a^	0.73 ± 0.07 ^ab^	0.78 ± 0.01 ^a^	43.90 ± 0.26 ^a^

ND—not detected. The mean values with different letters in the column in each group are statistically different (*p* < 0.05). “±” indicates standard deviation. G—grains, CS—control sprouts, PRS—probiotic-rich sprouts; AIN-L—liver of the AIN group, HFD-L—liver of the HFD group, HFDCS-L—liver of the HFDCS group, HFDPRS-L—liver of the HFDPRS group, AIN-F—feces of the AIN group, HFD-F—feces of the HFD group, HFDCS-F—feces of the HFDCS group, HFDPRS-F—feces of the AIN group, AIN—group fed with the basic diet AIN-93M; HFD—group fed with the basic diet AIN 93M with the addition of lard; HFDCS—group fed with the basic diet AIN-93M with the addition of lard and lyophilisate from control sprouts; HFDPRS—a group fed with the basic diet AIN-93M with the addition of lard and modified buckwheat sprouts lyophilisate, AIN-S—serum of rats of the AIN group, HFD-S—serum of rats of the HFD group, HFDCS-S—serum of rats of the HFDCS group, HFDPRS-S—serum of rats of the HFDPRS group.

**Table 4:** The content of fatty acids in buckwheat seeds (mg/g lipids) and sprouts (mg/g lipids), experimental diets (mg/g lipids), serum (µg/100 µL serum), liver (mg/g lipids) and feces (mg/g lipids).

Fatty Acids	G	CS	PRS	AIN	HFD	HFDCS	HFDPRS	AIN-S	HFD-S	HFDCS-S	HFDPRS-S	AIN-L	HFD-L	HFDCS-L	HFDPRS-L	AIN-F	HFD-F	HFDCS-F	HFDPRS-F
C10:0	ND	ND	ND	ND	ND	ND	ND	46.38 ± 3.12 ^b^	34.99 ± 2.34 ^a^	32.62 ± 2.21 ^a^	32.11 ± 2.49 ^a^	ND	ND	ND	ND	ND	ND	ND	ND
C14:0	ND	0.72 ± 0.02 ^a^	1.53 ± 0.06 ^c^	5.49 ± 0.15 ^a^	9.77 ± 0.39 ^b^	9.90 ± 0.24 ^b^	9.56 ± 0.30 ^b^	454.36 ± 3.09 ^c^	366.97 ± 5.75 ^a^	385.64 ± 5.81 ^b^	389.95 ± 4.97 ^b^	2.13 ± 0.16 ^ab^	1.82 ± 0.05 ^a^	2.51 ± 0.21 ^b^	2.30 ± 0.17 ^ab^	18.48 ± 0.00 ^c^	2.41 ± 0.23 ^a^	4.47 ± 0.54 ^b^	3.88 ± 0.69 ^b^
C 16:0	127.82 ± 0.20 ^c^	59.83 ± 0.11 ^a^	89.91 ± 0.10 ^b^	89.79 ± 0.93 ^a^	195.29 ± 0.35 ^b^	204.26 ± 0.25 ^d^	199.41 ± 0.32 ^c^	121.31 ± 4.06 ^c^	88.18 ± 4.50 ^a^	86.36 ± 4.77 ^a^	96.37 ± 2.93 ^b^	149.26 ± 4.63 ^a^	140.19 ± 6.48 ^a^	166.31 ± 3.27 ^b^	162.09 ± 2.68 ^b^	132.61 ± 0.00 ^c^	48.26 ± 0.86 ^a^	69.25 ± 4.13 ^b^	62.95 ± 7.22 ^b^
C18:0	22.52 ± 0.19 ^c^	10.83 ± 0.19 ^a^	16.63 ± 0.46 ^b^	44.69 ± 0.33 ^a^	111.89 ± 0.32 ^b^	118.23 ± 0.79 ^c^	111.89 ± 0.39 ^b^	84.95 ± 3.07 ^a^	92.13 ± 2.37 ^b^	85.62 ± 3.78 ^a^	93.16 ± 4.31 ^b^	133.68 ± 3.62 ^bc^	94.07 ± 4.37 ^a^	131.25 ± 3.49 ^b^	141.15 ± 0.86 ^c^	176.63 ± 0.00 ^c^	81.72 ± 1.57 ^a^	120.24 ± 9.68 ^b^	123.28 ± 4.72 ^b^
C20:0	11.66 ± 0.38 ^b^	7.92 ± 0.08 ^a^	12.36 ± 0.14 ^b^	2.95 ± 0.15 ^c^	1.59 ± 0.07 ^a^	2.14 ± 0.05 ^b^	2.01 ± 0.06 ^b^	ND	ND	ND	ND	ND	ND	ND	ND	ND	ND	3.82 ± 0.00 ^a^	4.05 ± 0.00 ^b^
C22:0	13.86 ± 0.27 ^b^	9.74 ± 0.23 ^a^	15.08 ± 0.12 ^c^	8.50 ± 0.52 ^b^	0.76 ± 0.05 ^a^	1.46 ± 0.11 ^a^	1.49 ± 0.05 ^a^	ND	ND	ND	ND	ND	ND	ND	ND	ND	ND	1.91 ± 0.00 ^a^	3.97 ± 0.00 ^b^
C23:0	ND	ND	ND	ND	ND	ND	ND	ND	ND	ND	ND	130.27 ± 2.77 ^a^	142.33 ± 2.33 ^b^	151.05 ± 1.80 ^b^	169.71 ± 4.79 ^c^	ND	ND	ND	ND
C24:0	7.47 ± 0.34 ^a^	7.16 ± 0.09 ^a^	10.38 ± 0.00 ^b^	2.96 ± 0.06 ^c^	0.31 ± 0.02 ^a^	0.64 ± 0.00 ^b^	0.59 ± 0.01 ^b^	1.99 ± 0.19 ^b^	1.47 ± 0.16 ^a^	1.79 ± 0.19 ^b^	1.51 ± 0.10 ^a^	ND	ND	ND	ND	ND	ND	ND	ND
C14:1	ND	ND	ND	ND	ND	ND	ND	22.58 ± 2.05 ^b^	20.65 ± 0.46 ^b^	17.98 ± 2.06 ^a^	16.56 ± 1.90 ^a^	ND	ND	ND	ND	ND	ND	ND	ND
C16:1	ND	1.59 ± 0.09 ^a^	2.24 ± 0.05 ^b^	1.75 ± 0.05 ^a^	13.63 ± 0.32 ^b^	13.64 ± 0.51 ^b^	13.29 ± 0.77 ^b^	11.83 ± 0.53 ^b^	3.71 ± 0.28 ^a^	4.08 ± 0.37 ^a^	4.04 ± 0.25 ^a^	4.23 ± 0.64 ^a^	12.75 ± 0.02 ^b^	5.19 ± 0.40 ^a^	4.87 ± 0.49 ^a^	ND	1.82 ± 0.25 ^b^	1.07 ± 0.24 ^ab^	0.66 ± 0.53 ^a^
C18:1	313.47 ± 0.32 ^c^	169.33 ± 2.13 ^a^	262.34 ± 0.44 ^b^	369.55 ± 0.35 ^d^	322.09 ± 0.23 ^a^	349.13 ± 0.82 ^c^	339.84 ± 0.44 ^b^	91.88 ± 3.73 ^c^	78.77 ± 2.44 ^ab^	75.32 ± 4.11 ^a^	83.52 ± 3.36 ^b^	174.32 ± 8.08 ^b^	112.57 ± 5.33 ^a^	180.56 ± 0.38 ^b^	170.17 ± 1.05 ^b^	40.76 ± 0.00 ^a^	91.88 ± 1.86 ^d^	74.96 ± 4.68 ^c^	56.72 ± 4.63 ^b^
C18:1 trans	ND	ND	ND	ND	ND	ND	ND	ND	ND	ND	ND	ND	ND	ND	ND	30.98 ± 0.00 ^c^	10.89 ± 1.00 ^ab^	10.99 ± 1.51 ^b^	8.21 ± 0.97 ^a^
C20:1	23.3 ± 0.07 ^b^	13.89 ± 0.69 ^a^	22.24 ± 0.28 ^b^	1.97 ± 0.09 ^a^	4.91 ± 0.14 ^b^	6.24 ± 0.03 ^d^	5.77 ± 0.00 ^c^	3.58 ± 0.07 ^b^	3.26 ± 0.24 ^a^	3.46 ± 0.27 ^ab^	3.95 ± 0.14 ^c^	2.51 ± 0.31 ^b^	ND	2.19 ± 0.08 ^b^	2.21 ± 0.09 ^b^	18.48 ± 0.00 ^c^	1.68 ± 0.23 ^a^	3.81 ± 0.23 ^b^	3.53 ± 0.38 ^b^
C 18:2	316.06 ± 0.89 ^c^	174.21 ± 1.89 ^a^	270.89 ± 0.67 ^b^	621.37 ± 1.90 ^c^	132.19 ± 0.38 ^a^	149.76 ± 0.05 ^b^	148.24 ± 0.19 ^b^	107.35 ± 4.07 ^c^	61.21 ± 1.73 ^a^	59.51 ± 3.73 ^a^	68.78 ± 2.53 ^b^	110.52 ± 7.09 ^a^	109.07 ± 3.73 ^ab^	126.39 ± 6.46 ^b^	122.07 ± 2.50 ^ab^	16.85 ± 0.00 ^d^	7.91 ± 0.53 ^b^	9.86 ± 0.79 ^c^	6.48 ± 0.29 ^a^
C18:3n-3	19.92 ± 0.10 ^c^	9.67 ± 0.43 ^a^	14.09 ± 0.08 ^b^	3.54 ± 0.01 ^a^	5.51 ± 0.23 ^b^	5.78 ± 0.19 ^b^	5.24 ± 0.12 ^b^	1.91 ± 0.21 ^b^	1.14 ± 0.15 ^a^	1.31 ± 0.22 ^a^	1.75 ± 0.10 ^b^	1.65 ± 0.32 ^b^	ND	1.39 ± 0.25 ^b^	1.21 ± 0.15 ^b^	ND	ND	ND	ND
C18:3n-6	ND	ND	ND	ND	ND	ND	ND	2.17 ± 0.17 ^c^	1.63 ± 0.15 ^b^	1.20 ± 0.14 ^a^	1.55 ± 0.12 ^b^	2.59 ± 0.12 ^a^	2.78 ± 0.09 ^ab^	2.85 ± 0.21 ^ab^	3.13 ± 0.20 ^b^	ND	ND	ND	ND
C20:3n-6	ND	ND	ND	ND	ND	ND	ND	1.55 ± 0.11 ^b^	1.05 ± 0.10 ^a^	1.50 ± 0.22 ^b^	1.49 ± 0.10 ^b^	ND	ND	ND	ND	ND	ND	ND	ND
C20:4n-6	ND	ND	ND	ND	ND	ND	ND	136.18 ± 5.08 ^b^	137.77 ± 3.54 ^b^	118.35 ± 4.35 ^a^	139.44 ± 4.23 ^b^	ND	ND	ND	ND	ND	ND	ND	ND
C22:5	ND	ND	ND	ND	ND	ND	ND	1.17 ± 0.06 ^a^	1.76 ± 0.15 ^c^	1.50 ± 0.15 ^b^	1.86 ± 0.11 ^c^	ND	ND	ND	ND	ND	ND	ND	ND
C22:6	ND	ND	ND	ND	ND	ND	ND	4.45 ± 0.35 ^a^	10.55 ± 0.34 ^d^	7.69 ± 0.46 ^b^	8.85 ± 0.50 ^c^	ND	ND	ND	ND	ND	ND	ND	ND

ND—not detected. The mean values with different letters in the row in each group are statistically different (*p* < 0.05). “±” indicates standard deviation. G—grains, CS—control sprouts, PRS—probiotic-rich sprouts; AIN-L—liver of the AIN group, HFD-L—liver of the HFD group, HFDCS-L—liver of the HFDCS group, HFDPRS-L—liver of the HFDPRS group, AIN-F—feces of the AIN group, HFD-F—feces of the HFD group, HFDCS-F—feces of the HFDCS group, HFDPRS-F—feces of the AIN group, AIN—group fed with the basic diet AIN-93M; HFD—group fed with the basic diet AIN 93M with the addition of lard; HFDCS—group fed with the basic diet AIN-93M with the addition of lard and lyophilisate from control sprouts; HFDPRS—a group fed with the basic diet AIN-93M with the addition of lard and modified buckwheat sprouts lyophilisate, AIN-S—serum of rats of the AIN group, HFD-S—serum of rats of the HFD group, HFDCS-S—serum of rats of the HFDCS group, HFDPRS-S—serum of rats of the HFDPRS group.

**Table 5:** The amount of individual groups of fatty acids (SFA, MUFA, PUFA) in buckwheat seeds (mg/g lipids) and sprouts (mg/g lipids), experimental diets (mg/g lipids), serum (µg/100 µL serum), liver (mg/g lipids) and feces (mg/g lipids).

	G	CS	PRS	AIN	HFD	HFDCS	HFDPRS	AIN-S	HFD-S	HFDCS-S	HFDPRS-S	AIN-L	HFD-L	HFDCS-L	HFDPRS-L	AIN-F	HFD-F	HFDCS-F	HFDPRS-F
**SFA**	183.33 ± 0.06 ^c^	96.20 ± 0.12 ^a^	145.89 ± 0.87 ^b^	154.38 ± 0.38 ^a^	319.61 ± 1.19 ^b^	336.63 ± 2.76 ^c^	324.95 ± 1.13 ^b^	708.99 ± 5.81 ^c^	583.74 ± 9.35 ^a^	592.03 ± 10.64 ^a^	613.10 ± 6.82 ^b^	415.34 ± 9.96 ^b^	378.41 ± 13.23 ^a^	451.12 ± 7.95 ^c^	475.25 ± 5.95 ^c^	327.72 ± 0.00 ^c^	132.39 ± 1.91 ^a^	199.69 ± 6.67 ^b^	198.13 ± 4.96 ^b^
**MUFA**	336.77 ± 0.25 ^c^	184.81 ± 2.91 ^a^	286.82 ± 0.21 ^b^	373.27 ± 0.48 ^d^	340.63 ± 0.70 ^a^	369.01 ± 1.35 ^c^	358.90 ± 1.21 ^b^	129.87 ± 3.27 ^c^	106.39 ± 2.24 ^b^	100.84 ± 4.86 ^a^	108.07 ± 3.54 ^b^	181.06 ± 8.91 ^b^	125.32 ± 5.35 ^a^	187.94 ± 0.11 ^b^	177.25 ± 1.43 ^b^	90.22 ± 0.00 ^b^	106.27 ± 3.16 ^c^	90.83 ± 5.99 ^b^	69.12 ± 5.89 ^a^
**PUFA**	335.98 ± 0.99 ^c^	183.88 ± 1.46 ^a^	284.98 ± 0.75 ^b^	624.91 ± 1.90 ^c^	137.70 ± 0.61 ^a^	155.54 ± 0.24 ^b^	153.48 ± 0.07 ^b^	254.78 ± 4.78 ^c^	215.11 ± 3.70 ^b^	191.06 ± 4.67 ^a^	223.72 ± 5.58 ^b^	114.76 ± 7.10 ^a^	111.85 ± 3.82 ^a^	130.63 ± 6.85 ^b^	126.41 ± 2.55 ^ab^	16.85 ± 0.00 ^d^	7.91 ± 0.53 ^b^	9.86 ± 0.79 ^c^	6.48 ± 0.29 ^a^

The mean values with different letters in the row in each group are statistically different (*p* < 0.05). “±” indicates standard deviation. G—grains, CS—control sprouts, PRS—probiotic-rich sprouts; AIN-L—liver of the AIN group, HFD-L—liver of the HFD group, HFDCS-L—liver of the HFDCS group, HFDPRS-L—liver of the HFDPRS group, AIN-F—feces of the AIN group, HFD-F—feces of the HFD group, HFDCS-F—feces of the HFDCS group, HFDPRS-F—feces of the AIN group, AIN—group fed with the basic diet AIN-93M; HFD—group fed with the basic diet AIN 93M with the addition of lard; HFDCS—group fed with the basic diet AIN-93M with the addition of lard and lyophilisate from control sprouts; HFDPRS—a group fed with the basic diet AIN-93M with the addition of lard and modified buckwheat sprouts lyophilisate, AIN-S—serum of rats of the AIN group, HFD-S—serum of rats of the HFD group, HFDCS-S—serum of rats of the HFDCS group, HFDPRS-S—serum of rats of the HFDPRS group.

**Table 6:** Composition of experimental diets (g/kg—AIN-93M, HFD diet; g/1.3kg—HFDCS, HFDPRS.

Component	AIN-93M	HFD ^1^	HFDCS ^2^	HFDPRS ^3^
Caseine	140	140	140	140
Soybean oil (rapeseed)	40	40	40	40
Wheat starch	622.5	422.5	422.5	422.5
Potato starch	50	50	50	50
Lard	-	200	200	200
Saccharose	100	100	100	100
Mineral mix	35	35	35	35
Vitamin mix	10	10	10	10
Choline	2.5	2.5	2.5	2.5
Control sprouts	-	-	300	-
Probiotic-rich sprouts	-	-	-	300

^1^ HFD—AIN93M + lard (high-fat diet). ^2^ HFDCS—high-fat diet + control sprouts. ^3^ HFDPRS—high-fat diet + probiotic-rich sprouts.

## Data Availability

Not applicable.

## Sample Availability

Samples of the compounds are not available from the authors.
